# Metabolic Disorders in HIV-Infected Adolescents Receiving Protease Inhibitors

**DOI:** 10.1155/2017/7481597

**Published:** 2017-02-15

**Authors:** Jeerunda Santiprabhob, Surapong Tanchaweng, Sirinoot Maturapat, Alan Maleesatharn, Watcharee Lermankul, Sirintip Sricharoenchai, Orasri Wittawatmongkol, Keswadee Lapphra, Wanatpreeya Phongsamart, Kulkanya Chokephaibulkit

**Affiliations:** ^1^Division of Endocrinology and Metabolism, Department of Pediatrics, Faculty of Medicine Siriraj Hospital, Mahidol University, Bangkok, Thailand; ^2^Division of Infectious Diseases, Department of Pediatrics, Faculty of Medicine Siriraj Hospital, Mahidol University, Bangkok, Thailand; ^3^Department of Pediatrics, Faculty of Medicine Siriraj Hospital, Mahidol University, Bangkok, Thailand

## Abstract

Protease inhibitor (PI) may cause abnormal glucose metabolism, abnormal lipid metabolism, and metabolic syndrome in HIV-infected adults but less well studied in Asian adolescents. This cross-sectional study evaluated anthropometric factors, oral glucose tolerance test, and lipid profiles of perinatally HIV-infected Thai adolescents who had received PI-based antiretroviral therapy for at least 6 months. Eighty adolescents were enrolled [median (IQR) age 16.7 (14.6–18.0) years, 42 males]. Metabolic syndrome, prediabetes, and type 2 diabetes mellitus (T2DM) were found in 8 (10%), 17 (22.1%), and 3 (3.8%) adolescents, respectively. Dyslipidemia was found in 56 (70%) adolescents, with hypertriglyceridemia being the most common type. In multivariate analysis, presence of lipohypertrophy (OR: 25.7, 95% CI: 3.2–202.8; *p* = 0.002) and longer duration of PI use (OR: 1.04, 95% CI: 1.00–1.08; *p* = 0.023) were associated with metabolic syndrome. Obesity (OR: 7.71, 95% CI: 1.36–43.7; *p* = 0.021), presence of lipohypertrophy (OR: 62.9, 95% CI: 4.97–795.6; *p* = 0.001), and exposure to stavudine for ≥6 months (OR: 8.18, 95% CI: 1.37–48.7; *p* = 0.021) were associated with prediabetes/T2DM, while exposure to tenofovir for ≥6 months reduced the risk (OR: 0.17, 95% CI: 0.04–0.78; *p* = 0.022). Metabolic disorders were commonly found in adolescents receiving PI. Careful monitoring and early intervention to modify cardiovascular risk should be systematically implemented in this population particularly those with exposure to stavudine.

## 1. Introduction

The number of perinatally HIV-infected children surviving into adolescence and adulthood has increased tremendously due to the accessibility and efficacy of highly active antiretroviral therapy (HAART). HAART prevents HIV-associated morbidity and mortality and has transformed HIV infection from a fatal disease into a chronic disease [1]. The current recommended initial HAART regimens generally consist of two nucleoside reverse transcriptase inhibitors (NRTIs) in combination with a nonnucleoside reverse transcriptase inhibitor (NNRTI) or a protease inhibitor (PI). Due to cost, NNRTI has been the main first-line regimen for children in resource-limited settings. About a quarter of adolescents in Asia that have grown up with HIV have had to switch from NNRTI regimens to boosted PI regimens, mostly as a result of imperfect adherence that led to treatment failure [2]. Lopinavir/ritonavir (LPV/r) has been the most often used PI in children, with high efficacy in virologic suppression and immunologic recovery. However, PIs [3] and some NRTIs, stavudine in particular, may cause long-term lipid and glucose metabolic complications [4–6].

PIs may cause abnormal glucose metabolism that ranges from prediabetic conditions, such as insulin resistance, impaired glucose tolerance (IGT), and impaired fasting glucose (IFG), to type 2 diabetes mellitus (T2DM). However, T2DM has rarely been reported in HIV-infected youth [7, 8], and the prediabetic conditions have not been routinely investigated. Dyslipidemia was commonly found among children receiving PI and may associate with lipodystrophy [6]. Incidence of dyslipidemia was found to be higher with LPV/r use than with darunavir and atazanavir use [9].

A condition that continues to develop relative to both prevalence and concern among clinicians is metabolic syndrome, which is defined in adults as the combination of central obesity with 2 or more of the following: hypertriglyceridemia, hypertension, IFG or T2DM, and low level of high-density lipoprotein-cholesterol (HDL-cholesterol) [10]. This condition was found to associate with development of T2DM and cardiovascular diseases (CVD) [11]. Although obesity has not been common among HIV-infected children and adolescents [12, 13], other features of metabolic syndrome, low HDL-cholesterol and hypertriglyceridemia were commonly found in this population [14, 15]. HIV-infected adults have an increased risk of CVD, as compared to the general population [16]. Dyslipidemia, abnormal glucose metabolism, HIV infection itself, and increased inflammatory cytokines are contributing factors to early CVD in HIV-infected patients [17]. It is possible that the risk of CVD may be greater in perinatally HIV-infected adolescents than in individuals who acquired infection in adulthood, since these patients were treated with HAART from very young age. It is, therefore, important to evaluate and modify the risks of developing early CVD starting from a young age in this patient population.

Although metabolic complications among HIV-infected adolescents have been previously studied, the reported findings were relatively heterogeneous [7, 8, 14, 15, 18, 19]. Moreover, data regarding the pathophysiology and prevalence of metabolic disorders, particularly metabolic syndrome and glucose metabolism disorder from the effects of HAART, in Asian population remains unclear and very limited. Accordingly, the aim of this study was to investigate the prevalence of metabolic complications among perinatally HIV-infected adolescents receiving stable PI-based HAART and to evaluate factors associated with the development of these metabolic complications.

## 2. Materials and Methods

### 2.1. Patients and Study Design

This cross-sectional study was conducted at Siriraj Hospital, a tertiary care center in Bangkok, during November 2012 through April 2014. Perinatally HIV-infected adolescents receiving PI-based HAART were enrolled. Inclusion criteria were as follows: (1) age older than 10 years; (2) exposure to PI-based HAART, which included NRTI, for at least 6 months; and (3) absence of any metabolic diseases, such as T2DM, hypertension (HT), or dyslipidemia, before starting PI-based HAART. After receiving parental written informed consent and patient assent to participate, patients were scheduled for a single study visit to undergo the following study processes and procedures: risk factors interview (smoking, alcohol consumption, and family history of metabolic diseases or CVD); physical examination; oral glucose tolerance test (OGTT); and lipid profile measurement. Medical records were reviewed for demographic and HIV-related data, including previous and current HAART, nadir and current CD4 counts, current viral load, and clinical stage according to US Centers for Disease Control and Prevention (CDC) staging system [20]. Due to toxicity and availability of safer alternative drugs at our site, stavudine and indinavir were discontinued from use in mid-2011.

Physical examination included vital signs, routine organ system examination, anthropometric measurements, and assessment of puberty, lipodystrophy, and presence of acanthosis nigricans. Systolic and diastolic blood pressures were measured after the patient rested for at least 5 minutes. Height was measured by Harpenden Stadiometer (Holtain Limited, Wales, United Kingdom) without shoes, and body weight was measured by digital scale (Tanita Corporation, Tokyo, Japan). Waist circumference was measured without clothing using a Gulick II tape measure at the midpoint between the 12th rib and the iliac crest at the end of expiration with the patient in a standing position.

Body mass index (BMI) calculation was weight (kg) divided by height^2^ (m). Percentage weight-for-height was determined using the following formula: (actual weight/ideal weight) × 100, using Thai standard growth curve reference [21]. Waist-to-height ratio calculation was waist circumference (cm) divided by height (cm).

Patient pubertal status was defined, as follows. Patients were regarded as being prepubertal if female breast development was Tanner stage I and if male testes were < 4 mL. Patients were considered to be in puberty if female breast development was ≥Tanner stage II and if male testes were ≥ 4 mL.

Patients were classified as having lipodystrophy if they had lipoatrophy, lipohypertrophy, or combined type, according to the European Pediatric Lipodystrophy Group [22]. Children with at least one sign of peripheral lipoatrophy (loss of subcutaneous fat at face, arms, legs, or buttocks) and at least one sign of central lipohypertrophy (increased abdominal girth, buffalo hump, or breast enlargement) were defined as combined type. Diagnosis of lipodystrophy was made by two investigators who agreed on the findings. Severity of lipodystrophy was defined as subtle (noticeable only if specifically looked for); moderate (easily noticeable by parent, patient, and/or physician); and severe (obvious to other observers) [23]. Presence or absence of acanthosis nigricans was evaluated. Observation for acanthosis nigricans was made at the neck and axilla area.

Patients were instructed to fast for 12 hours overnight in preparation for the OGTT and serum lipid profile. Patients were given glucose 1.75 g/kg, with a maximum of 75 g. Venous blood samples for glucose and insulin were collected before and at 1 and 2 hours after glucose was given to the patient. Blood investigations included total cholesterol, triglycerides, HDL-cholesterol, low-density lipoprotein-cholesterol (LDL-cholesterol), glycated hemoglobin (HbA1c), CD4 cell count, and HIV RNA viral load. Glucose was measured with an automated analyzer (Cobas c system, Roche Diagnostic, Manheim, Germany). Insulin was analyzed with a biochemical autoanalyzer (Cobas e, Roche Diagnostic). HbA1c was determined by turbidimetric inhibition immunoassay (Roche Diagnostic). Total cholesterol, HDL-cholesterol, LDL-cholesterol, and triglycerides were analyzed with a biochemical autoanalyzer (Cobas c system, Roche Diagnostic). LDL-cholesterol was calculated, unless triglycerides were ≥400 mg/dL, in which case LDL-cholesterol was measured directly. CD4 cell count was determined by flow cytometry using direct immunofluorescence method (BD Tritest CD3 FITC/CD4 PE/CD45 PerCP; BD Biosciences, San Jose, CA, USA). Plasma HIV RNA load was measured by real-time polymerase chain reaction (PCR), with the limit of detection at 40 copies/mL using Abbott platform (Abbott Molecular Inc., Des Plaines, IL, USA).

The protocol for this study was approved by Siriraj Hospital Research Ethics Committee.

### 2.2. Definitions

Metabolic syndrome was defined according to the consensus statement of the International Diabetes Federation (IDF) [10, 24], with modifications in the definition for central obesity and abnormal glucose metabolism. Due to the lack of a national standard for waist circumference and BMI percentile, we used waist-to-height ratio ≥ 0.5 to define central obesity [25, 26]. Metabolic syndrome was diagnosed if patients had central obesity plus 2 or more of the following criteria: (1) triglycerides level ≥ 1.7 mmol/L; (2) HDL-cholesterol level < 1.03 mmol/L for patients younger than 16 years of age and HDL-cholesterol level < 1.29 mmol/L in female and < 1.03 mmol/L in male patients 16 years of age or older; (3) systolic blood pressure (SBP) ≥ 130 mmHg or diastolic blood pressure (DBP) ≥ 85 mmHg; and (4) fasting plasma glucose (FPG) ≥ 5.6 mmol/L or having T2DM [10, 24] or impaired glucose tolerance (IGT).

Abnormal OGTT was defined using American Diabetes Association (ADA) criteria [27]. Impaired fasting glucose (IFG) was defined as FPG of 5.6–6.9 mmol/L. IGT was defined as 2-hour plasma glucose (2-hour PG) of 7.8–11.0 mmol/L. T2DM was defined as FPG ≥ 7.0 mmol/L or 2-hour PG ≥ 11.1 mmol/L. IFG and IGT were considered prediabetes. Insulin resistance was defined using the homeostasis model assessment of insulin resistance (HOMA-IR) [fasting insulin (*μ*U/mL) × FPG (mmol/L)/22.5]. Insulin resistance was diagnosed when the HOMA-IR score was ≥ 2.5 in prepubertal or ≥ 4.0 in pubertal children [28].

Hypertriglyceridemia and low HDL-cholesterol were defined according to the IDF consensus statement for metabolic syndrome [10, 24]. High LDL-cholesterol was defined as LDL-cholesterol level ≥ 3.4 mmol/L [29].

### 2.3. Statistical Analysis

Categorical variables are expressed as number and percentage. Continuous variables are presented using median and interquartile range (IQR) due to the nonparametric characteristics of the data. Independent two-group comparisons of continuous variables and categorical variables were performed using Mann–Whitney *U* test and *χ*^2^ or Fisher's exact test, respectively. Potential factors associated with prediabetes/T2DM, lipid disorders, and metabolic syndrome, including obesity, lipodystrophy/lipohypertrophy/lipoatrophy, CDC stage, nadir CD4 cell count, current CD4 cell count, current viral load, duration of PIs and HAART, and exposure to each antiretroviral agent, were analyzed in univariate logistic regression analysis. Factors identified as significant in univariate analysis (*p* < 0.2) and potential confounding factors (age or Tanner stage, gender, and family history of T2DM and/or lipid disorder) were entered into step-wise multivariate logistic regression analysis to identify factors associated with each evaluated disorder. Odds ratio (OR) and 95% confidence interval (CI) are shown for each factor in univariate analysis and each statistically significant factor in multivariate analysis. Cumulative duration of exposure and/or exposure of a certain drug for ≥ 6 months were used to test for association between each antiretroviral drug and metabolic disorders. If both variables were found to be significantly related to a metabolic disorder in univariate analysis, exposure of that drug ≥ 6 months was included in multivariate analysis. Data were analyzed using STATA version 11.2 (StataCorp, LP, College Station, TX, USA). A *p* value of < 0.05 was regarded as being statistically significant.

## 3. Results

Eighty HIV-infected adolescents were enrolled. The median (IQR) age was 16.7 (14.6–18.0) years and 42 (52.5%) were male. Seventy-five patients (93.7%) were in puberty. On the study evaluation day, 64 (80.0%) patients had an undetectable viral load, and the median (IQR) CD4 cell count was 656 (525.5–815.5) cells/mm^3^. Family history of CVD was reported in 2 (2.5%) patients, hypertension in 19 (23.8%), T2DM in 24 (30.0%), and dyslipidemia in 19 (23.8%). Obesity and acanthosis nigricans was found in 15 (18.8%) and 11 (13.8%) patients, respectively. Twenty-six (32.5%) patients had lipodystrophy, with lipoatrophy found in 18 (22.5%), lipohypertrophy in 4 (5.0%), and combined type in 4 (5%). All lipoatrophy and lipohypertrophy were moderate in severity. Demographic, anthropometric, and metabolic characteristics are shown in Table 1.

The median (IQR) duration of HAART and PI treatment was 114.3 (78.8–129.6) months and 72.6 (56.1–81.2) months, respectively. Forty-eight (60%) patients were currently receiving LPV/r, 24 (30%) were receiving atazanavir/ritonavir, and 8 (10%) were receiving darunavir/ritonavir. For NRTI, 55 (68.8%) were currently receiving tenofovir, 22 (27.5%) receiving zidovudine, and 79 (98.8%) receiving lamivudine. Details relating to HAART exposure are shown in Table 2.

### 3.1. Prevalence of Abnormal Glucose Metabolism, Abnormal Lipid Metabolism, and Metabolic Syndrome

Overt T2DM was found in 3 pubertal patients (3.8%; 2 females and 1 male) and all had obesity. One patient had combined type lipodystrophy and another had lipohypertrophy. Prediabetes was found in 17 (22.1%) patients and insulin resistance was found in 27 (33.8%).

Dyslipidemia was commonly found, with 70% of patients having at least one type of dyslipidemia. Hypertriglyceridemia was the most common dyslipidemia found (43.8%), followed by low HDL-cholesterol (38.8%).

Overall, 8 (10%) obese subjects fully met the criteria for metabolic syndrome. Another 16 (20%) adolescents without obesity had at least 2 features of metabolic syndrome. Prevalence of metabolic disorders is shown in Table 3.

### 3.2. Factors Associated with Metabolic Syndrome

Patients with metabolic syndrome had significantly longer median (IQR) duration of indinavir exposure than patients without metabolic syndrome [42.8 (16.9–53.6)* versus* 0 (0–32.2) months; OR: 1.03, 95% CI: 1.0–1.06; *p* = 0.020]. Moreover, all patients with metabolic syndrome and only 44.4% of patients without metabolic syndrome had received indinavir for ≥ 6 months. Since all patients with metabolic syndrome had been on indinavir for at least 6 months, univariate analysis could not be performed. Fisher's exact test was used instead and the result showed significant association between exposure to indinavir for at least 6 months and metabolic syndrome (*p* = 0.005).

In univariate analysis, factors associated with metabolic syndrome were older age (OR: 1.4, 95% CI: 1.07–1.90; *p* = 0.014), presence of lipohypertrophy (OR: 17, 95% CI: 3.1–94.4; *p* = 0.001), and longer duration of PI use (OR: 1.027, 95% CI: 1.001–1.05; *p* = 0.038). In multivariate analysis, presence of lipohypertrophy (OR: 25.7, 95% CI: 3.2–202.8; *p* = 0.002) and longer duration of PI use (OR: 1.04, 95% CI: 1.00–1.08; *p* = 0.023) were shown to be independent risk factors for metabolic syndrome. Analysis of factors associated with metabolic syndrome is shown in Table 4.

### 3.3. Factors Associated with Prediabetes/T2DM

In univariate analysis, factors associated with having prediabetes/T2DM were obesity (OR: 5.0, 95% CI: 1.5–16.6; *p* = 0.008), presence of acanthosis nigricans (OR: 7.5, 95% CI: 1.9–29.6; *p* = 0.004), lipohypertrophy (OR: 31.8, 95% CI: 3.6–280.9; *p* = 0.002), lipodystrophy (OR: 3.7, 95% CI: 1.3–10.6; *p* = 0.016), and exposure to indinavir for at least 6 months (OR: 3.05, 95% CI: 1.03–9.0; *p* = 0.044). In multivariate analysis, obesity (OR: 7.71, 95% CI: 1.36–43.7; *p* = 0.021), presence of lipohypertrophy (OR: 62.9, CI: 4.97–795.6; *p* = 0.001), and exposure to stavudine treatment for at least 6 months (OR: 8.18, 95% CI: 1.37–48.7; *p* = 0.021) were significantly associated with prediabetes/T2DM. In contrast, exposure to tenofovir for at least 6 months significantly reduced the risk of having prediabetes/T2DM (OR: 0.17, 95% CI: 0.04–0.78; *p* = 0.022). Analysis of factors associated with prediabetes/T2DM is shown in Table 5.

### 3.4. Factors Associated with Dyslipidemia

In multivariate analysis, factors associated with hypertriglyceridemia were increased age (OR: 1.28, 95% CI: 1.02–1.61; *p* = 0.032), nadir CD4 cell count > 350 cells/mm^3^ (OR: 7.56, 95% CI: 1.68–34.03; *p* = 0.008), and presence of lipodystrophy (OR: 3.48, 95% CI: 1.01–12.01; *p* = 0.048). Longer duration of efavirenz exposure was modestly associated with high LDL-cholesterol (OR: 1.02, 95% CI: 1.00–1.03; *p* = 0.048), while exposure to tenofovir ≥ 6 months decreased the risk of high LDL-cholesterol (OR: 0.25, 95% CI: 0.07–0.93; *p* = 0.039). Finally, older age was associated with low HDL-cholesterol (OR: 1.35, 95% CI: 1.08–1.69; *p* = 0.009), while exposure to didanosine ≥ 6 months (OR: 0.08, 95% CI: 0.02–0.43; *p* = 0.003) and being male (OR: 0.16, 95% CI: 0.05–0.57; *p* = 0.005) decreased the risk of having low HDL-cholesterol.

## 4. Discussion

Metabolic complications contribute to CVD and T2DM in HIV-infected patients exposed to PIs [30]. These conditions result in mortality and morbidity among patients with well-controlled HIV disease. In this study of 80 perinatally HIV-infected adolescents, we found that 10% of the adolescents receiving PIs had complete pictures of metabolic syndrome, 20% had two or more features of metabolic syndrome without obesity, and 70% had any form of dyslipidemia. From the results of OGTT, we found prediabetes/T2DM in 26% of patients and 34% had insulin resistance. With careful anthropometric measurement and physical examination, we found that 32% had lipodystrophy, with most cases having peripheral lipoatrophy. We also found duration of PI use (indinavir in particular) and lipohypertrophy to be associated with metabolic syndrome, and lipohypertrophy, obesity, and prolonged stavudine exposure to be associated with prediabetes/T2DM. Based on our review of the literature this is the first comprehensive metabolic study in perinatally HIV-infected adolescents in the region.

HIV-infected children and adolescents were found to be smaller and thinner than healthy children [12, 13]; obesity was found to be uncommon. Unexpectedly, we found that 19% of our HIV-infected adolescents met our criteria for obesity (waist-to-height ratio ≥ 0.5). A study in Thai HIV-infected adults found an overall prevalence of metabolic syndrome of 22.2%, with patients with antiretroviral therapy (ART) exposure having a significantly higher prevalence of metabolic syndrome than ART-naïve patients [31]. Several other studies reported prevalence rates that varied from 7–46% [32–37]. Differences in study populations, ethnicities, host genetic factors, antiretroviral class/type of PIs, and variability in diagnostic criteria all contribute to variations in prevalence. Many studies in adults used NCEP criteria [38] that require 3 of 5 risk factors (obesity, high glucose, low HDL-cholesterol, high triglyceride, and high blood pressure), eliminating the absolute requirement of obesity as a diagnostic criterion [36, 37]. In this study, a third of adolescents had two or more metabolic syndrome features with or without obesity. Ethnic background may play an important role of relatively high prevalence of metabolic syndrome features found in our cohort. As previously shown in a study among Thai adults, the prevalence of metabolic syndrome was 32.6% (using NCEP III criteria) [39], higher than that found among American adults which reported the prevalence of 22.9% [40]. This high prevalence of metabolic syndrome features necessitates preventive intervention for CVD in our population.

The studies that have investigated factors associated with metabolic syndrome in adults found and reported mixed results. A large study from Spain in 710 HIV-infected adults found exposure to LPV/r and stavudine to be significantly associated with metabolic syndrome [41]. Another large study in 477 American adults found use of LPV/r, didanosine, increasing viral load, and higher BMI to be associated with metabolic syndrome [42]. The Women's Interagency HIV study, which included 1,725 seropositive and 668 seronegative women, reported that having HIV infection, older age, higher BMI, current smoking, and use of stavudine were associated with metabolic syndrome, while ritonavir-boosted PIs were not [43]. In our study, we found exposure to indinavir (but not LPV/r) and lipohypertrophy (but not lipoatrophy) to be associated with metabolic syndrome. Other studies found lipohypertrophy to be associated with indinavir use [44, 45]. Given that indinavir was discontinued in our patients in mid-2011, a strong argument can be made suggesting that this agent has long-lasting metabolic effect, even after being discontinued.

Our cohort, which consisted mainly of pubertal adolescents, had a higher prevalence of prediabetes/T2DM than previously reported studies from various regions [7, 8, 46]. A study from Spain in 99 adolescents with perinatally acquired HIV infection (median age: 15.3 years) reported a prevalence of insulin resistance of 30.6% and IFG of only 4.6% [7]. Two studies in American children using OGTT assessment found that children and adolescents who grew up with HIV had insulin resistance in 12% of prepubertal and 16–33% in pubertal patients, IGT in at least 15% of patients, and no patients had T2DM [8, 46]. A study in young Latin American children (mean ± standard deviation age: 7.5 ± 2.1 years) rarely found IFG (only 1 child) and found insulin resistance in only 6.8% [18], which was relatively close to the 10% rate of insulin resistance found in prepubertal African children [14]. The relatively high prevalence of prediabetes/T2DM found in our study may be attributable to the genetic predisposition of Asians to develop T2DM [47–49]. Our prevalence finding was similar to the 27.5% prediabetes finding from a study in 149 HIV-infected Thai adults, 92% of whom were receiving ART [50]. A large study in Thai adults found that Thai population was susceptible to development of cardiovascular risk conditions (e.g., T2DM and hypertension) even with mild degree of obesity [51].

The pathogenesis of insulin resistance in HIV-infected individuals could be multifactorial, including certain antiretroviral agents, chronic hepatitis C coinfection, altered fat distribution, mitochondrial dysfunction, elevated proinflammatory cytokines, genetic predisposition, and other general risk factors, like advancing age, high BMI, and male gender [52–54]. Insulin resistance caused by antiretroviral agents occurs by 2 mechanisms: direct interference with insulin signaling and/or as a consequence of mitochondrial dysfunction in adipocytes and/or muscle associated with lipodystrophy [54]. PIs cause abnormal glucose metabolism by inhibiting GLUT4, a glucose transporter, which results in decreased glucose transport into muscle and adipose tissue [55, 56]. Antiretroviral agents that have been shown to associate with abnormal glucose and lipid metabolism and lipodystrophy in HIV-infected adults are old generation PIs, such as indinavir and ritonavir, and thymidine NRTIs, such as stavudine, zidovudine, and didanosine [52–55, 57, 58]. Indinavir has been shown to inhibit insulin-stimulated glucose uptake into adipocytes in an animal model [55] and caused insulin resistance after 4 weeks of exposure in healthy subjects [59]. The Data Collection on Adverse Events of Anti-HIV Drugs (D:A:D) study, a very large prospective observational study of 33,389 HIV-infected patients, found that stavudine had the strongest relationship with T2DM, with exposure to zidovudine and didanosine found to associate with an increased risk of T2DM [58]. A preliminary analysis in the D:A:D study also found that current indinavir exposure was an additional risk factor for T2DM [58]. The Multicenter AIDS Cohort Study (MACS) found cumulative exposure to NRTIs associated with an increased risk of hyperinsulinemia, with no similar finding observed for cumulative exposure to NNRTIs or PIs [57].

Very few studies in HIV-infected children have reported association between certain antiretroviral agents and risk of having abnormal glucose metabolism; however, many studies found obesity and lipodystrophy associated with insulin resistance [7, 8, 46, 60]. Association between PI use and abnormal glucose metabolism has been controversial in pediatric studies [8, 12]. Based on the results of our study, abdominal obesity, presence of lipohypertrophy, and exposure to stavudine treatment for at least 6 months were associated with risk of having prediabetes/T2DM. Stavudine was used in most resource-limited settings during the early era of HAART, and 64% of our cohort had previously received stavudine. We stopped prescribing stavudine at our center in mid-2011. Despite its discontinuation, we found that it might contribute to prediabetes/T2DM, independent of the permanent lipoatrophy caused by its use. In our study, exposure to tenofovir decreased the risk of having abnormal glucose level, consistent with a study that reported no change in insulin sensitivity in healthy volunteers after exposure to tenofovir [61].

Dyslipidemia, particularly hypertriglyceridemia, was commonly found in HIV-infected children and adolescents, with a prevalence range of 35–52% [7, 12, 15, 18, 46, 62]. Many factors contribute to dyslipidemia in HIV-infected patients, including non-HIV-related factors (e.g., diet, obesity, sedentary lifestyle, and family history), HIV infection itself, and certain antiretroviral agents [9]. Ritonavir has been associated with hypertriglyceridemia and hypercholesterolemia [9, 63, 64]. LPV/r was more likely to induce dyslipidemia than atazanavir/ritonavir and darunavir/ritonavir [9]. Moreover, stavudine has been associated with increased level of total cholesterol, LDL-cholesterol, and triglyceride levels, as well as the development of lipoatrophy [6, 9, 65]. Among NNRTI agents, efavirenz has been associated with mild increase in triglycerides and LDL-cholesterol, as well as increase in HDL-cholesterol level [9, 66]. A study in 156 African children found LPV/r-based ART induced low HDL-cholesterol and high total cholesterol, LDL-cholesterol, and triglyceride level, as compared to nevirapine [19]. A study among prepubertal African children found recent efavirenz exposure and cumulative LPV/r exposure to be associated with increased LDL-cholesterol level [14].

Based on the results of our study, increasing age, nadir CD4 cell count > 350 cells/mm^3^, and presence of lipodystrophy increased the risk of developing hypertriglyceridemia. In contrast to other studies, we did not find association between LPV/r or other PI use and hypertriglyceridemia [7, 15, 62]. Our findings were consistent with a study in prepubertal African children that did not find association between LPV/r-based or efavirenz-based ART and high triglyceride level [14].

In our study, we found that high LDL-cholesterol was associated with duration of efavirenz exposure, while exposure to tenofovir longer than 6 months decreased the risk, which was consistent with previous reports [64, 67, 68]. Again, in contrast to previous studies, we did not find association between high LDL-cholesterol and ritonavir or LPV/r exposure [12, 14, 19].

With regard to low HDL-cholesterol, we found that older age was a risk factor, while being male and exposure to didanosine for longer than 6 months were protective factors. Risk factors for having low HDL-cholesterol varied in different studies [7, 14, 46]. In contrast to our finding, a study in children and adolescents in Spain found that male gender and large abdominal conference were associated with low HDL-cholesterol level [7]. Didanosine has been reported to have an intermediate unfavorable effect on lipid disorder [9]. A large adult cohort in the D:A:D study found that recent exposure to NRTI, abacavir, and didanosine was associated with increased myocardial infarction risk [69]. In contrast and from a cross-sectional substudy of the Women's Interagency HIV Study, didanosine, lamivudine, nevirapine, and efavirenz were independently associated with higher HDL-cholesterol [70]. Taken together, factors associated with each type of dyslipidemia were found to be inconsistent among studies, suggesting different multifactorial effects in each setting.

This study has some mentionable limitations. First, we defined metabolic syndrome using IDF criteria; however, we do not have standard waist circumference reference for Thai youth. Instead, we used waist-to-height ratio ≥ 0.5 to define abdominal obesity, which could be less accurate. Second, some of our patients underwent many HAART regimen switches, which makes assessing the effect of a single antiretroviral agent difficult. However, we used both cumulative duration of drug exposure and drug exposure ≥ 6 months to evaluate the effect of antiretroviral agents on metabolic disorder.

Although our study has a relatively small sample size, we performed OGTT in every patient, which is not routinely done. OGTT discovered a substantial number of patients with glucose metabolism disorder and a number of patients with metabolic syndrome. These important findings alert us to be aware of metabolic complications among this patient population. The very limited available options of antiretroviral drugs in children made the switching to lipid/metabolic friendlier drugs very difficult. Darunavir, the most lipid/metabolic friendly PI, was only recently available in the country for children. This denunciates the problem with not enough antiretroviral drugs approved for HIV-infected children.

At present, treatments for dyslipidemia in HIV-infected children are quite limited. Statin is only approved in children older than 10 years of age, while fibrate is not recommended in children. Statin toxicities include elevated liver and muscle enzymes, and combination use with certain ARTs may increase the risk of developing these side effects [71]. HIV-infected youth who developed T2DM should be treated with metformin or insulin. If metformin is used in combination with NRTI, lactate level needs to be monitored [71]. Another potent insulin sensitizer, thiazolidinedione, has not been approved in children and may cause elevated liver enzymes. In this regard, the metabolic complications found among our study cohort may not have been optimally treated.

## 5. Conclusion

Metabolic features, abnormal glucose metabolism, and dyslipidemia were commonly found in adolescents receiving PIs. Subtle metabolic disorders that occur at a young age have the potential of leading to early CVD and adversely affecting long-term prognosis. Screening and preventive procedures should be implemented in routine care of adolescents receiving PI-based HAART, particularly those with exposure to stavudine.

## Figures and Tables

**Table 1:** Demographic, anthropometric, and metabolic characteristics of 80 HIV-infected adolescents receiving protease inhibitor- (PI-) based highly active antiretroviral therapy (HAART).

Patients characteristics	*N* = 80
Age (years), median (IQR)	16.7 (14.6–18.0)
Male, *n* (%)	42 (52.5)
History of smoking, *n* (%)	2 (2.5)
History of alcohol consumption, *n* (%)	3 (3.8)
Family history of CVD, *n* (%)	2 (2.5)
Family history of HT, *n* (%)	19 (23.8)
Family history of T2DM, *n* (%)	24 (30.0)
Family history of dyslipidemia, *n* (%)	19 (23.8)
Percentage weight-for-age (%), median (IQR)	89.4 (79.5–99.8)
Percentage weight-for-height (%), median (IQR)	101.2 (89.1–110.6)
BMI (kg/m^2^), median (IQR)	18.3 (16.3–20.4)
Waist circumference (cm), median (IQR)	66.9 (62–72.7)
Waist-to-height ratio, median (IQR)	0.44 (0.41–0.48)
Obesity (waist-to-height ratio ≥0.5), *n* (%)	15 (18.8)
SBP (mmHg), median (IQR)	114 (106–120)
DBP (mmHg), median (IQR)	70 (64–75)
Acanthosis nigricans, *n* (%)	11 (13.8)
Lipodystrophy, *n* (%)	26 (32.5)
(i) Lipoatrophy, *n* (%)	18 (22.5)
(ii) Lipohypertrophy, *n* (%)	4 (5.0)
(iii) Combined type, *n* (%)	4 (5.0)
CDC stage, *n* (%)	
(i) Severely symptomatic (Stage C), *n* (%)	25 (31.3)
(ii) Nonsevere stage, *n* (%)	55 (68.7)
Nadir CD4 cell count (cells/mm^3^) (*n* = 79)	
(i) <100 cells/mm^3^, *n* (%)	31 (39.2)
(ii) 100–350 cells/mm^3^, *n* (%)	26 (32.9)
(iii) >350 cells/mm^3^, *n* (%)	22 (27.9)
CD4 cell count (cells/mm^3^), median (IQR)	656 (525.5–815.5)
(i) <350 cells/mm^3^, *n* (%)	10 (12.5)
(ii) 350–500 cells/mm^3^, *n* (%)	7 (8.7)
(iii) >500 cells/mm^3^, *n* (%)	63 (78.8)
Viral load (copies/mL)	
(i) ≤40 copies/mL, *n* (%)	64 (80.0)
(ii) >40 copies/mL, *n* (%)	16 (20.0)
Total cholesterol (mmol/L), median (IQR)	4.39 (3.96–5.26)
Triglycerides (mmol/L), median (IQR)	1.51 (1.14–2.31)
LDL-cholesterol (mmol/L), median (IQR)	2.42 (2.06–2.99)
HDL-cholesterol (mmol/L), median (IQR)	1.19 (1.01–1.41)
FPG (mmol/L), median (IQR)	4.66 (4.38–4.97)
2-hour PG (mmol/L), median (IQR)	6.66 (5.61–7.44)
Fasting insulin (pmol/L), median (IQR)	95 (62–154)
2-hour insulin (pmol/L), median (IQR)	642 (376–1686)
HOMA-IR, median (IQR)	2.8 (1.8–4.4)
HbA1c (%), median (IQR)	5.2 (4.9–5.5)

IQR, interquartile range; CVD, cardiovascular disease; T2DM, type 2 diabetes mellitus; HT, hypertension; BMI, body mass index; SBP, systolic blood pressure; DBP, diastolic blood pressure; CDC, Centers for Disease Control; LDL, low-density lipoprotein; HDL, high-density lipoprotein; FPG, fasting plasma glucose; PG, plasma glucose.

**Table 2:** Antiretroviral treatment exposure of 80 HIV-infected adolescents receiving protease inhibitor- (PI-) based highly active antiretroviral therapy (HAART).

HAART exposure	*N* = 80
Current HAART regimen, *n* (%)	
(i) 2NRTI + boosted PI	72 (90.0)
(ii) NRTI + NNRTI + boosted PI	8 (10.0)
Antiretroviral drugs currently being received, *n* (%)	
(i) Abacavir	1 (1.3)
(ii) Didanosine	3 (3.8)
(iii) Lamivudine	79 (98.8)
(iv) Tenofovir	55 (68.8)
(v) Zidovudine	22 (27.5)
(vi) Efavirenz	11 (13.8)
(vii) Nevirapine	1 (1.3)
(viii) Lopinavir/ritonavir	48 (60.0)
(ix) Atazanavir/ritonavir	24 (30.0)
(x) Darunavir/ritonavir	8 (10.0)
Duration of PI (months), median (IQR)	72.6 (56.1–81.2)
Duration of HAART (months), median (IQR)	114.3 (78.8–129.6)
Cumulative duration of antiretroviral drug ever received (months), median (IQR)^*∗*^	
(i) Didanosine	29.8 (0–65.3)
(ii) Stavudine	21.1 (0–51.3)
(iii) Tenofovir	20.0 (3.8–41.4)
(iv) Zidovudine	59.7 (31.3–82.6)
(v) Efavirenz	13.8 (0–35.8)
(vi) Nevirapine	0.4 (0–28.8)
(vii) Lopinavir/ritonavir	51.5 (28.7–71.7)
(viii) Atazanavir	0 (0–8.8)
(ix) Darunavir	0 (0-0)
(x) Indinavir	6.3 (0–37.2)
(xi) Full dose ritonavir	0 (0-0)
Number of patients ever received the antiretroviral drug for ≥ 6 months, *n* (%)	
(i) Didanosine	56 (70.0)
(ii) Stavudine^*∗∗*^	51 (63.7)
(iii) Tenofovir	57 (71.3)
(iv) Zidovudine	73 (91.3)
(v) Efavirenz	43 (53.7)
(vi) Nevirapine	34 (42.5)
(vii) Lopinavir/ritonavir	79 (98.8)
(viii) Atazanavir	23 (28.8)
(ix) Darunavir	13 (16.3)
(x) Indinavir^*∗∗*^	40 (50.0)
(xi) Full dose Ritonavir	8 (10.0)

^*∗*^Includes those not receiving the drug.

^*∗∗*^Stavudine and indinavir were no longer in use after mid-2011.

NRTI, nucleoside reverse transcriptase inhibitor; NNRTI, nonnucleoside reverse transcriptase inhibitor; IQR, interquartile range.

**Table 3:** Prevalence of abnormal glucose metabolism, lipid metabolism, and metabolic syndrome features in HIV-infected adolescents receiving protease inhibitor- (PI-) based highly active antiretroviral therapy (HAART).

Diagnosis	*N* (%)
Abnormal glucose metabolism	34 (42.5)
(i) Prediabetes	17 (22.1)
IGT	15 (19.5)
IFG	2 (2.6)
(ii) Diabetes	3 (3.8)
(iii) Insulin resistance	27 (33.8)
Abnormal lipid metabolism	56 (70.0)
(i) Hypertriglyceridemia	35 (43.8)
(ii) High LDL-cholesterol	17 (21.3)
(iii) Low HDL-cholesterol	31 (38.8)
High blood pressure	7 (8.8)
Metabolic syndrome	8 (10.0)
(i) Obesity plus 2 metabolic syndrome features	6 (7.5)
(ii) Obesity plus 3 metabolic syndrome features	2 (2.5)
Metabolic syndrome features without obesity	16 (20.0)
(i) 2 metabolic syndrome features	14 (17.5)
(ii) 3 metabolic syndrome features	2 (2.5)

IGT, impaired glucose tolerance; IFG, impaired fasting glucose.

**Table 4:** Clinical characteristics of patients and risk factors for metabolic syndrome in HIV-infected adolescents receiving protease inhibitor- (PI-) based highly active antiretroviral therapy (HAART).

Characteristics and factors	Metabolic syndrome (*n* = 8)	Nonmetabolic syndrome (*n* = 72)	Univariate analysis		Multivariate analysis^*∗*^	
			Crude OR (95% CI)	*p* value	Adjusted OR (95% CI)	*p* value
*Demographics and anthropometrics*						
Age (years), median (IQR)	19.1 (16.4–21.4)	16.5 (14.3–17.6)	1.4 (1.07–1.9)	0.014		
Gender, *n* (%)						
Male	5 (62.5)	37 (51.4)	1.6 (0.3–7.1)	0.553		
Female	3 (37.5)	35 (48.6)	1			
Family history of CVD or HT, *n* (%)						
Yes	3 (37.5)	17 (23.6)	1.9 (0.4–8.9)	0.396		
No	5 (62.5)	55 (76.4)	1			
Family history of T2DM, *n* (%)						
Yes	4 (50.0)	20 (27.8)	2.6 (0.6–11.4)	0.205		
No	4 (50.0)	52 (72.2)	1			
Family history of dyslipidemia, *n* (%)						
Yes	3 (37.5)	16 (22.2)	2.1 (0.4–9.7)	0.344		
No	5 (62.5)	56 (77.8)	1			
Waist circumference (cm), median (IQR)	82 (80.3–94.7)	66.2 (61.2–70.1)	1.26 (1.1–1.4)	0.001		
BMI (kg/m^2^), median (IQR)	23.4 (22.3–26.9)	17.6 (16.1–19.4)	2.1 (1.3–3.4)	0.001		
Percentage weight-for-height (%), median (IQR)	128.7 (119.6–139.4)	98.5 (87.8–106.1)	1.15 (1.06–1.24)	<0.001		
Waist-to-height ratio, median (IQR)	0.54 (0.51–0.58)	0.43 (0.39–0.46)	1.46 × 10^17^ (2.72 × 10^17^ to 7.88 × 10^26^)	0.001		
Obesity, *n* (%)	8 (100)	7 (9.7)	—	<0.001^*∗∗*^		
SBP (mmHg), median (IQR)	125 (119–129)	113 (105–119)	1.18 (1.05–1.32)	0.004		
DBP (mmHg), median (IQR)	77 (68–88)	69 (64–73)	1.12 (1.02–1.22)	0.018		
High blood pressure, *n* (%)						
Yes	3 (37.5)	4 (5.6)	10.2 (1.8–58.7)	0.009		
No	5 (62.5)	68 (94.4)	1			
Acanthosis nigricans, *n* (%)	5 (62.5)	6 (8.3)	18.3 (3.5–96.2)	0.001		
Lipoatrophy, *n* (%)	1 (12.5)	21 (29.2)	0.3 (0.04–3.0)	0.336		
Lipohypertrophy, *n* (%)	4 (50.0)	4 (5.6)	17 (3.1–94.4)	0.001	25.7 (3.2–202.8)	0.002
Lipodystrophy, *n* (%)	4 (50.0)	22 (30.6)	2.3 (0.5–9.9)	0.275		
Tanner staging, *n* (%)						
Pre-pubertal stage	0 (0.0)	5 (6.9)	—	—		
Pubertal stage	8 (100.0)	67 (93.1)				
*Metabolic parameters*						
Total cholesterol (mmol/L), median (IQR)	5.58 (4.53–6.95)	4.34 (3.90–5.04)	1.03 (1.007–1.04)	0.005		
Triglycerides (mmol/L), median (IQR)	4.44 (1.88–5.03)	1.48 (1.06–2.19)	1.005 (1.00–1.009)	0.032		
LDL-cholesterol (mmol/L), median (IQR)	2.57 (2.27–4.13)	2.40 (2.05–2.93)	1.022 (0.99–1.046)	0.057		
HDL-cholesterol (mmol/L), median (IQR)	1.05 (0.91–1.11)	1.22 (1.02–1.45)	0.95 (0.89–1.01)	0.107		
FPG (mmol/L), median (IQR)	5.00 (4.44–7.41)	4.63 (4.39–4.91)	1.06 (1.00–1.12)	0.050		
2-hour PG (mmol/L), median (IQR)	8.77 (7.16–13.15)	6.58 (5.50–7.30)	1.03 (1.0–1.06)	0.007		
Fasting insulin (pmol/L), median (IQR)	177 (142–304)	91 (58–131)	1.02 (0.99–1.04)	0.157		
2-hour insulin (pmol/L), median (IQR)	1793 (790–3552)	586 (376–1463)	1.002 (1.0–1.004)	0.049		
HOMA-IR, median (IQR)	6.2 (5.0–8.9)	2.7 (1.7–4.0)	1.1 (1.0–1.2)	0.048		
HbA1c (%), median (IQR)	5.4 (5.2–6.9)	5.2 (4.9–5.5)	3.8 (0.9–16.2)	0.072		
*HIV-specific disease characteristic*						
CDC stage, *n* (%)						
Severely symptomatic (Stage C)	3 (37.5)	22 (30.6)	1.36 (0.3–6.2)	0.689		
Nonsevere stage	5 (62.5)	50 (69.4)	1			
Nadir CD4 cell counts (cells/mm^3^), median (IQR)	107.5 (35.5–244)	184 (33–395)	0.99 (0.99–1.00)	0.348		
<100 cells/mm^3^, *n* (%)	3 (37.5)	28 (39.4)	1			
100–350 cells/mm^3^, *n* (%)	4 (50.0)	22 (31.0)	1.7 (0.3–8.4)	0.516		
>350 cells/mm^3^, *n* (%)	1 (12.5)	21 (29.6)	0.4 (0.04–4.6)	0.496		
CD4 cell counts (cells/mm^3^), median (IQR)	682 (490–832)	656 (525.5–802)	0.99 (0.997–1.001)	0.664		
<350 cells/mm^3^, *n* (%)	1 (12.5)	9 (12.5)	1			
350–500 cells/mm^3^, *n* (%)	1 (12.5)	6 (8.3)	1.5 (0.08–28.9)	0.788		
>500 cells/mm^3^, *n* (%)	6 (75.0)	57 (79.2)	0.95 (0.1–8.8)	0.962		
Viral load (copies/mL), median (IQR)^*∗∗∗*^	40 (40-40)	40 (40-40)	0.99 (0.99–1.00)	0.731		
≤40 copies/mL, *n* (%)	7 (87.5)	57 (79.2)	1.8 (0.2–16.2)	0.581		
>40 copies/mL, *n* (%)	1 (12.5)	15 (20.8)	1			
Duration of PIs (months), median (IQR)	89.3 (70.4–123.0)	72.3 (53.2–80.0)	1.027 (1.001–1.05)	0.038	1.04 (1.00–1.08)	0.023
Duration of HAART (months), median (IQR)	126.7 (90.7–129.1)	106.5 (78.8–129.7)	1.007 (0.98–1.03)	0.546		
Ever received didanosine ≥ 6 months, *n* (%)	6 (75.0)	50 (69.4)	1.32 (0.25–7.1)	0.746		
Ever received stavudine ≥ 6 months, *n* (%)	7 (87.5)	44 (61.1)	4.4 (0.5–38.2)	0.173	9.5 (0.5–165.4)	0.122
Ever received tenofovir ≥ 6 months, *n* (%)	6 (75.0)	51 (70.8)	1.2 (0.2–6.6)	0.805		
Ever received zidovudine ≥ 6 months, *n* (%)	8 (100.0)	65 (90.3)	—	—		
Ever received efavirenz ≥ 6 months, *n* (%)	4 (50.0)	39 (54.2)	0.8 (0.2–3.6)	0.823		
Ever received nevirapine ≥ 6 months, *n* (%)	3 (37.5)	31 (43.1)	0.8 (0.18–3.6)	0.763		
Ever received lopinavir/ritonavir ≥ 6 months, *n* (%)	8 (100.0)	71 (98.6)	—	—		
Ever received atazanavir ≥ 6 months, *n* (%)	4 (50.0)	19 (26.4)	2.8 (0.6–12.3)	0.175		
Ever received darunavir ≥ 6 months, *n* (%)	3 (37.5)	10 (13.9)	3.7 (0.8–18.1)	0.103		
Ever received indinavir ≥ 6 months, *n* (%)	8 (100.0)	32 (44.4)	—	0.005^*∗∗*^		
Ever received full dose ritonavir ≥ 6 months, *n* (%)	1 (12.5)	7 (9.7)	1.3 (0.14–12.4)	0.804		

^*∗*^Input variables: age, gender, family history of T2DM, family history of dyslipidemia, PI duration, lipohypertrophy, and exposure to stavudine ≥6 months.

^*∗∗*^Fisher's exact test. ^*∗∗∗*^Viral load ≤ 40 copies/mL was expressed as 40 in the statistical analysis.

OR, odds ratio; CI, confidence interval; IQR, interquartile range; CVD, cardiovascular disease; HT, hypertension; T2DM, type 2 diabetes mellitus; SBP, systolic blood pressure; DBP, diastolic blood pressure; CDC, Centers for Disease Control; FPG, fasting plasma glucose; PG, plasma glucose.

**Table 5:** Clinical characteristics of patients and risk factors for prediabetes and type 2 diabetes mellitus in HIV-infected adolescents receiving protease inhibitor- (PI-) based highly active antiretroviral therapy (HAART).

Characteristics and factors	Prediabetes/T2DM (*n* = 20)	Normal glucose level (*n* = 60)	Univariate analysis		Multivariate analysis^*∗*^	
			Crude OR (95% CI)	*p *value	Adjusted OR (95% CI)	*p* value
*Demographics and anthropometrics*						
Age (years), median (IQR)	16.9 (14.5–19.1)	16.6 (14.6–17.8)	1.04 (0.9–1.2)	0.603		
Gender, *n* (%)						
Male	13 (65.0)	29 (48.3)	2.0 (0.7–5.7)	0.200	4.16 (0.87–19.78)	0.073
Female	7 (35.0)	31 (51.7)	1		1	
Family history of T2DM, *n* (%)						
Yes	7 (35.0)	17 (28.3)	1.4 (0.5–4.0)	0.574		
No	16 (65.0)	43 (71.7)	1			
Waist circumference (cm), median (IQR)	75.25 (61–82)	66.65 (63.1–70.1)	1.06 (1.01–1.12)	0.027		
BMI (kg/m^2^), median (IQR)	19.3 (15.7–23.4)	17.8 (16.5–19.7)	1.18 (1.01–1.37)	0.035		
Percentage weight-for-height (%), median (IQR)	107.3 (96.7–128.5)	98.5 (87.8–107.7)	1.03 (1.00–1.07)	0.028		
Waist-to-height ratio, median (IQR)	0.48 (0.41–0.53)	0.44 (0.40–0.46)	7210.9 (1.1–4.71 × 10^7^)	0.047		
Obesity, *n* (%)	8 (40.0)	7 (11.7)	5.0 (1.5–16.6)	0.008	7.71 (1.36–43.7)	0.021
High blood pressure, *n* (%)						
Yes	4 (20.0)	3 (5.0)	4.8 (0.96–23.44)	0.056		
No	16 (80.0)	57 (95.0)	1			
Acanthosis nigricans, *n* (%)	7 (35.0)	4 (6.7)	7.5 (1.9–29.6)	0.004		
Lipoatrophy, *n* (%)	8 (40.0)	14 (23.3)	2.2 (0.7–6.4)	0.153		
Lipohypertrophy, *n* (%)	7 (35.0)	1 (1.7)	31.8 (3.6–280.9)	0.002	62.9 (4.97–795.6)	0.001
Lipodystrophy, *n* (%)	11 (55.0)	15 (25.0)	3.7 (1.3–10.6)	0.016		
Tanner staging, *n* (%)						
Pre-pubertal stage	2 (10.0)	3 (5.0)	1			
Pubertal stage	18 (90.0)	57 (95.0)	0.5 (0.1–3.1)	0.433		
*Metabolic parameters*						
Total cholesterol (mmol/L), median (IQR)	4.66 (4.12–5.97)	4.35 (3.90–4.99)	1.01 (1.0–1.02)	0.030		
Triglycerides (mmol/L), median (IQR)	1.70 (1.24–3.35)	1.48 (1.03–2.72)	1.0 (0.99–1.00)	0.075		
LDL-cholesterol (mmol/L), median (IQR)	2.34 (2.06–3.73)	2.43 (2.05–2.87)	1.0 (0.99–1.02)	0.218		
HDL-cholesterol (mmol/L), median (IQR)	1.17 (1.05–1.36)	1.19 (0.10–1.42)	1.0 (0.97–1.02)	0.997		
FPG (mmol/L), median (IQR)	5.02 (4.50–5.52)	4.60 (4.39–4.88)	1.12 (1.03–1.22)	0.007		
2-hour PG (mmol/L), median (IQR)	8.82 (8.05–9.94)	6.11 (5.33–6.88)	1.19 (1.08–1.31)	0.001		
Fasting insulin (pmol/L), median (IQR)	156 (70–301)	90 (55–122)	1.05 (1.01–1.08)	0.012		
2-hour insulin (pmol/L), median (IQR)	1611 (812–3775)	543 (357–1058)	1.005 (1.002–1.008)	0.001		
HOMA-IR, median (IQR)	5.0 (2.8–10.1)	2.6 (1.6–3.6)	1.26 (1.06–1.49)	0.007		
HbA1c (%), median (IQR)	5.2 (4.9–5.5)	5.2 (4.9–5.5)	1.9 (0.7–5.2)	0.205		
*HIV-specific disease characteristic*						
CDC stage, *n* (%)						
Severely symptomatic (stage C)	5 (25.0)	20 (33.3)	0.7 (0.2–2.1)	0.488		
Nonsevere stage	15 (75.0)	40 (66.7)	1			
Nadir CD4 cell count (cells/mm^3^), median (IQR)	188.5 (36–392.5)	161 (33–389)	1.00 (0.99–1.00)	0.851		
<100 cells/mm^3^, *n* (%)	6 (30.0)	25 (42.4)	1			
100–350 cells/mm^3^, *n* (%)	8 (40.0)	18 (30.5)	1.8 (0.5–6.3)	0.322		
>350 cells/mm^3^, *n* (%)	6 (30.0)	16 (27.1)	1.6 (0.4–5.7)	0.499		
CD4 cell count (cells/mm^3^), median (IQR)	766.5 (597–936.5)	624 (502–753.5)	1.00 (0.99–1.00)	0.121	1.002 (0.99–1.00)	0.061
<350 cells/mm^3^, *n* (%)	1 (5.0)	9 (15.0)	1			
350–500 cells/mm^3^, *n* (%)	1 (5.0)	6 (10.0)	1.5 (0.1–28.9)	0.788		
>500 cells/mm^3^, *n* (%)	18 (90.0)	45 (75.0)	3.6 (0.4–30.5)	0.240		
Viral load (copies/mL), median (IQR)^*∗∗*^	40 (40-40)	40 (40-40)	1.0 (1.0–1.0)	0.475		
≤40 copies/mL, *n* (%)	18 (90.0)	46 (76.7)	2.7 (0.6–13.3)	0.211		
>40 copies/mL, *n* (%)	2 (10.0)	14 (23.3)	1			
Duration of PIs (months), median (IQR)	73.8 (58.6–82.1)	72.5 (50.8–81.2)	1.00 (0.99–1.02)	0.389		
Duration of HAART (months), median (IQR)	119.9 (82.1–128.1)	106.2 (77.0–130.9)	1.00 (0.99–1.02)	0.609		
Ever received didanosine ≥ 6 months, *n* (%)	15 (75.0)	41 (68.3)	1.4 (0.4–4.4)	0.574		
Ever received stavudine ≥ 6 months, *n* (%)	16 (80.0)	35 (58.3)	2.8 (0.8–9.6)	0.089	8.18 (1.37–48.7)	0.021
Ever received tenofovir ≥ 6 months, *n* (%)	11 (55.0)	46 (76.7)	0.37 (0.13–1.08)	0.069	0.17 (0.04–0.78)	0.022
Ever received zidovudine ≥ 6 months, *n* (%)	20 (100.0)	53 (88.3)	—	—		
Ever received efavirenz ≥ 6 months, *n* (%)	9 (45.0)	34 (56.7)	0.6 (0.2–1.7)	0.367		
Ever received nevirapine ≥ 6 months, *n* (%)	10 (50.0)	24 (40.0)	1.5 (0.5–4.1)	0.435		
Ever received lopinavir/ritonavir ≥ 6 months, *n* (%)	20 (100.0)	59 (98.3)	—	—		
Ever received atazanavir ≥ 6 months, *n* (%)	8 (40.0)	15 (25.0)	2 (0.7–5.8)	0.204		
Ever received darunavir ≥ 6 months, *n* (%)	5 (25.0)	8 (13.3)	2.2 (0.6–7.6)	0.228		
Ever received indinavir ≥ 6 months, *n* (%)	14 (70.0)	26 (43.3)	3.05 (1.03–9.0)	0.044	2.6 (0.68–10.3)	0.159
Ever received full dose ritonavir ≥ 6 months, *n* (%)	1 (5.0)	7 (11.7)	0.4 (0.05–3.4)	0.404		

^*∗*^Input variables: gender, Tanner stage, family history of T2DM, obesity, lipohypertrophy, current CD4 cell count, exposure to stavudine for at least 6 months, exposure to tenofovir for at least 6 months, and exposure to indinavir for at least 6 months. ^*∗∗*^Viral load ≤ 40 copies/mL was expressed as 40 in the statistical analysis.

OR, odds ratio; CI, confidence interval; IQR, interquartile range; T2DM, type 2 diabetes mellitus; SBP, systolic blood pressure; DBP, diastolic blood pressure; CDC, Centers for Disease Control; FPG, fasting plasma glucose; PG, plasma glucose.
