# Correction to “Metabolic Disorders in HIV‐Infected Adolescents Receiving Protease Inhibitors”

**DOI:** 10.1155/bmri/9814702

**Published:** 2026-11-11

J. Santiprabhob, S. Tanchaweng, S. Maturapat, A. Maleesatharn, W. Lermankul, S. Sricharoenchai, O. Wittawatmongkol, K. Lapphra, W.Phongsamart, and K. Chokephaibulkit, “Metabolic Disorders in HIV‐Infected Adolescents Receiving Protease Inhibitors”, *BioMed Research International*, vol. 2017 (2017). https://doi.org/10.1155/2017/7481597.

In the article titled “Metabolic Disorders in HIV‐Infected Adolescents Receiving Protease Inhibitors”, there was an error in section 2.1 related to the duration of the study described in the article. This error was introduced by the authors during manuscript preparation and should be corrected as follows:

[“This cross‐sectional study was conducted at Siriraj Hospital, a tertiary care center in Bangkok, during November 2012 through April 2014.”]

should read

This cross‐sectional study was conducted at Siriraj Hospital, a tertiary care center in Bangkok, from October 2013 until November 2014.

The authors have provided the corresponding ethics approval document and research protocol, which confirm the above‐mentioned study duration.

We apologise for these errors.

